# Phlegmonous duodenitis in an immunocompromised patient

**DOI:** 10.1002/deo2.212

**Published:** 2023-02-02

**Authors:** Daisuke Miyagishima, Naoto Fujita, Hiromasa Suzuki

**Affiliations:** ^1^ Department of Gastroenterology and Hepatology Numazu City Hospital Shizuoka Japan

**Keywords:** duodenitis, *Escherichia coli*, immunocompromised host, phlegmonous duodenitis, *Streptococcus parasanguinis*

## Abstract

Phlegmonous duodenitis is an extremely rare condition, and only a few cases have been previously reported. Here, we report a case of phlegmonous duodenitis caused by *Streptococcus parasanguinis* and *Escherichia coli* in a 78‐year‐old immunocompromised patient with diabetes mellitus and immunosuppressive drugs. Abdominal computed tomography showed diffuse thickening of the duodenum and gastric antrum, and esophagogastroduodenoscopy revealed some erosions with purulent discharge and reddish and edematous mucosa in the duodenal bulb. A bacteriological culture test detected the two abovementioned bacteria and established the diagnosis of phlegmonous duodenitis. Following the initiation of antibiotic treatment, his condition rapidly improved. Endoscopists should be aware of this rare entity and pay attention to the endoscopic duodenal findings similar to those of phlegmonous gastritis, particularly in immunocompromised patients who develop abdominal symptoms with severe inflammation.

## INTRODUCTION

Among infectious diseases in the gastrointestinal tract, cases of phlegmonous gastritis have been accumulated and summarized in the previous reports^1^, ^2^, ^3^, ^4^, ^5^, ^6^, ^7^; however, phlegmonous duodenitis is an extremely rare condition in the clinical setting, and its natural course and imaging findings remain unknown to date. We herein present a case of PD caused by *Streptococcus parasanguinis* (*S. parasanguinis*) and *Escherichia coli* (*E. coli*) in an immunocompromised patient with diabetes mellitus and immunosuppressive drugs. This case report highlights the characteristic findings of PD, provides clues for diagnosis, and points out the way to manage this rare disease.

## CASE REPORT

**FIGURE 1:**
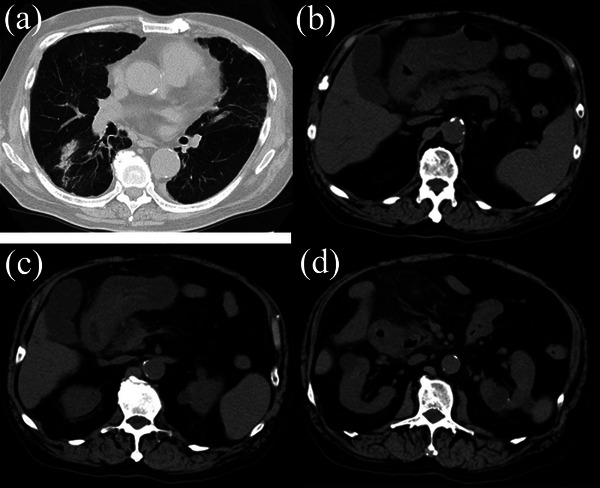
Computed tomography of the chest and abdomen. (a) Computed tomography of the chest reveals patchy opacification in the lower lobe of the right lung. This finding remains unchanged and is consistent with the previous diagnosis of cryptogenic organizing pneumonia. (b–d) Computed tomography of the abdomen shows significantly diffuse thickening of the gastric antrum and duodenum with fat stranding around these organs.

**FIGURE 2:**
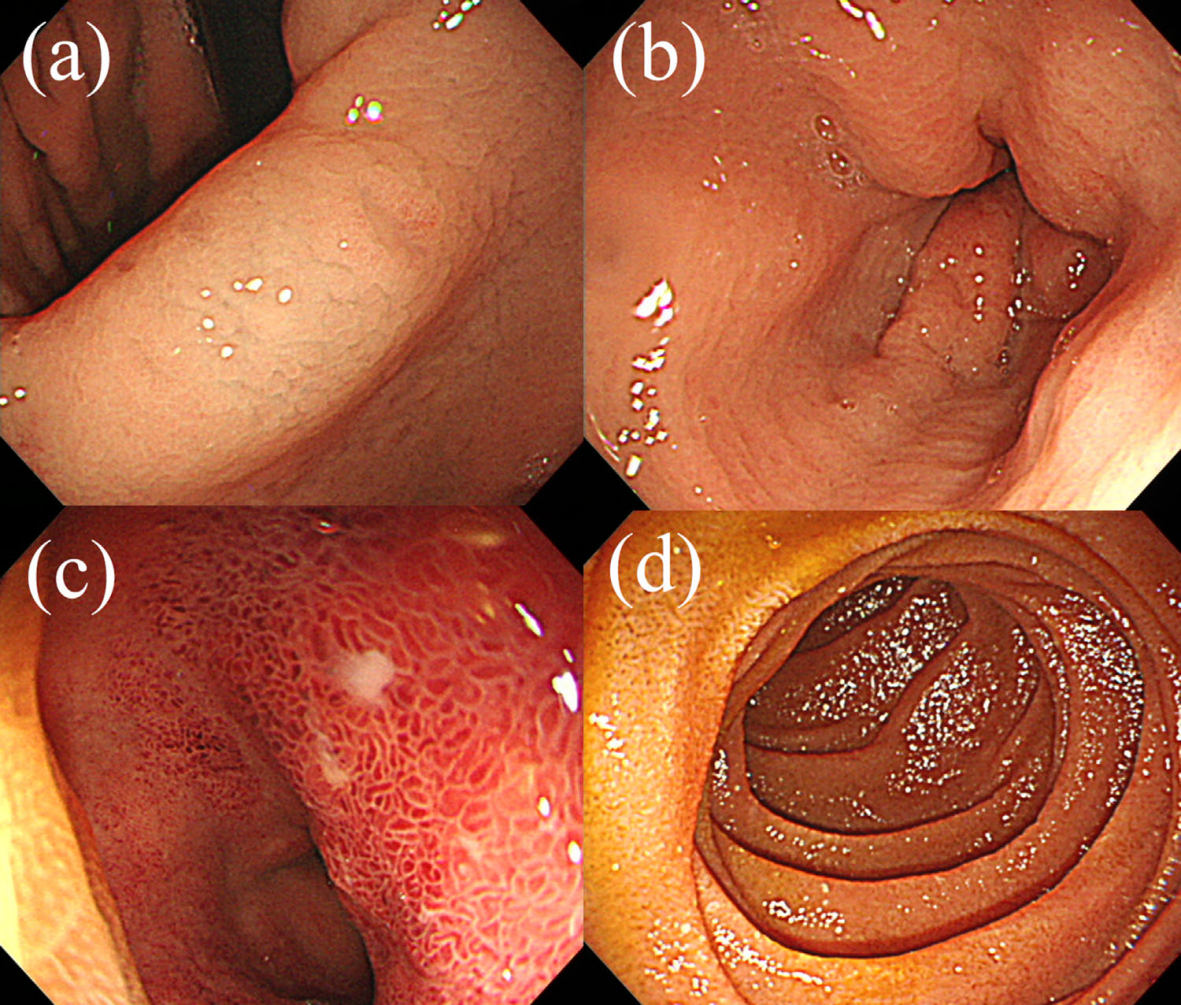
Esophagogastroduodenoscopy findings. (a) The gastric angle is edematous without any ulcerations. (b) The gastric antrum is also edematous; however, no erosions and ulcers specific to the acute gastric and duodenal mucosal lesion are noted. (c) The duodenal bulb shows some small erosions containing a small amount of pus with markedly reddish and edematous mucosa. (d) The descending part of the duodenum shows no remarkable mucosal change.

A 78‐year‐old man with rheumatoid arthritis and interstitial pneumonia was admitted to our hospital due to abdominal pain and vomiting for 1 week. He described stinging epigastric pain accompanied by continuous nausea; however, he denied any diarrhea, melena, or hematochezia. Except for heumatoid arthritis and interstitial pneumonia, his past medical history included hypertension, steroid‐induced diabetes mellitus, drug‐induced thrombocytopenia, and aspiration pneumonia, which was successfully treated with doripenem (DRPM) 3 months before admission. He had been taking tacrolimus (1.5 mg/day), prednisolone (11 mg/day), salazosulfapyridine, voglibose, eldecalcitol, amlodipine, irbesartan, trimethoprim‐sulfamethoxazole, and magnesium oxide. He was an ex‐smoker who smoked 20 cigarettes daily until the age of 60; however, he denied drinking habits and any recent history of traveling abroad. On admission, he was afebrile with a temperature of 36.8°C, and his blood pressure and heart rate were 96/65 mmHg and 79 beats/min, respectively. His oxygen saturation was 98% while breathing 1.5 L/min of oxygen via nasal cannula. Physical examination revealed a slightly distended abdomen and mild tenderness in the epigastric region without guarding or rebound tenderness. The following were his laboratory data: white blood cell count, 6900/mm^3^; hemoglobin, 10.1 g/dl; platelet count, 34,000/mm^3^; albumin, 2.6 g/dl; amylase, 37 U/L; C‐reactive protein, 19.67 mg/dl; procalcitonin, 0.96 ng/ml; β‐D‐glucan, <6.0 pg/ml; *Helicobacter pylori* antibody titer, 12.7 U/ml; KL‐6, 529 U/ml; HbA1c, 7.7%; and human immunodeficiency virus antibody, negative. Urine analysis was unremarkable except for mild urinary protein (+1). Initial abdominal computed tomography (CT) revealed significant diffuse thickening of the gastric antrum and the first and second portions of the duodenum as well as fat stranding around these organs (Figure 1). Based on his clinical symptoms and these radiological findings on CT, we made a tentative diagnosis of acute gastric and duodenal mucosal lesion. After his admission, esophagogastroduodenoscopy (EGD) was performed. However, contrary to our initial prediction, EGD revealed a mucosal defect with a small amount of pus and significantly reddish and edematous mucosa in the duodenal bulb (Figure 2c). The mucosa of the gastric angle and antrum was edematous; however, no mucosal lesions in the stomach were noted (Figure 2a,b). Since the findings of the duodenal bulb seemed completely different from those of acute gastric and duodenal mucosal lesion, although similar to those of phlegmonous gastritis, a mucosal culture of the duodenal bulb was obtained in addition to biopsy specimens of the duodenum and gastric antrum for pathological examination.

**FIGURE 3:**
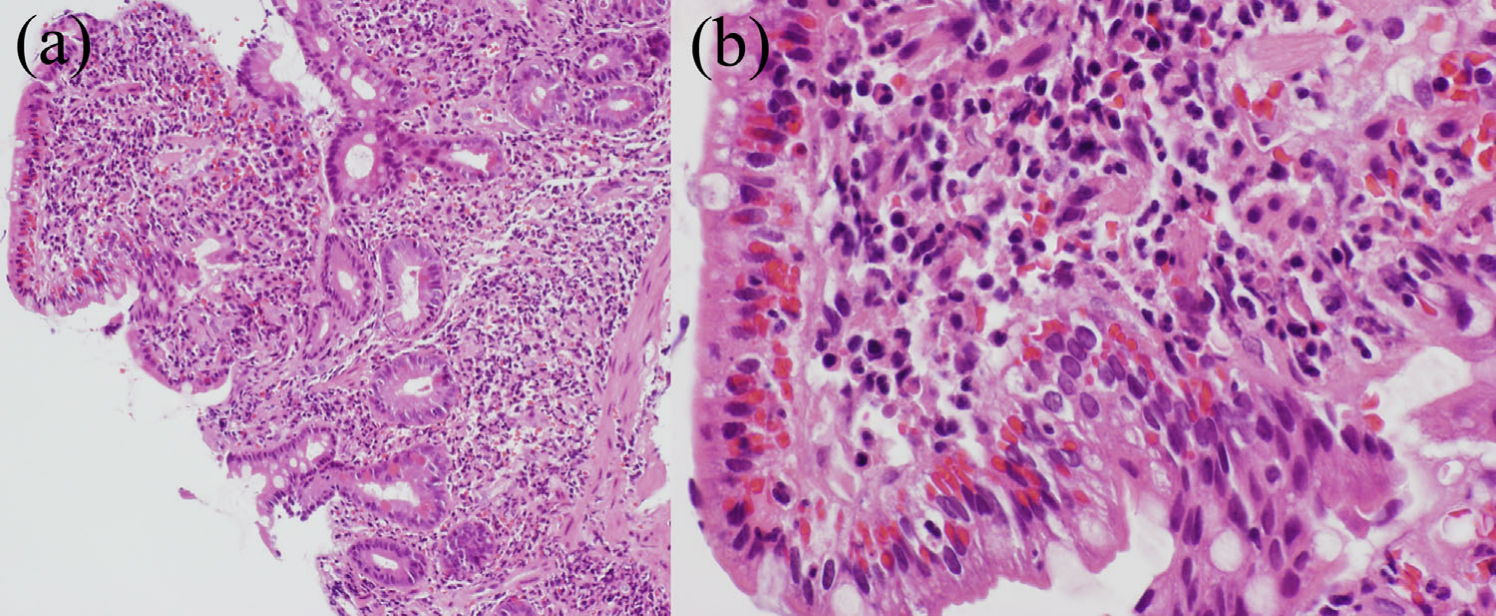
Pathological examination of the specimen obtained from the duodenal bulb. (a) Duodenal biopsy reveals a significant neutrophil leukocytic infiltrate in the edematous lamina propria (100**×**). (b) Severe hemorrhage is observed on the surface of the duodenal mucosa (400**×**). These findings are nonspecific although consistent with those of gastrointestinal infectious diseases.

**FIGURE 4:**
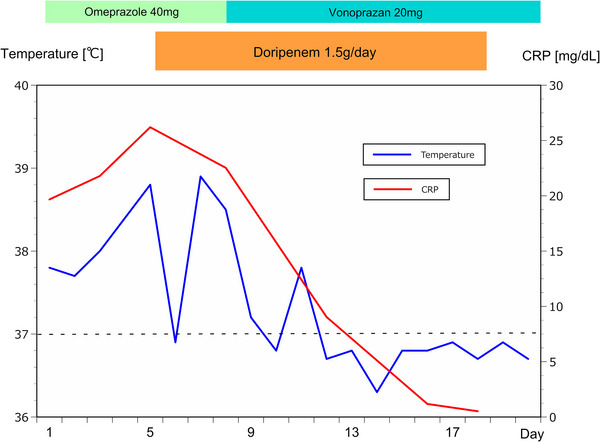
The patient's clinical course. Following the initiation of antibiotic therapy with doripenem on day 5, the patient's temperature and serum C‐reactive protein (CRP) level gradually decreased. The patient's gastrointestinal symptoms quickly resolved, and he was discharged home from our hospital on day 20.

Following this endoscopic examination, the patient was started with intravenous saline infusion and omeprazole injection (20 mg twice a day) under the provisional diagnosis of duodenitis. Despite these conservative therapies, his abdominal discomfort and high fever persisted, and the C‐reactive protein level continued to increase up to 26.20 mg/dl on day 5. The culture of the duodenal mucosa was positive for the growth of *S. parasanguinis* and *E. coli* (extended‐spectrum beta‐lactamase‐producing *E. coli*). Histopathological examination of the biopsied duodenal mucosa demonstrated a diffuse neutrophil leukocytic infiltrate, severe hemorrhage, and edema of the lamina propria (Figure 3), while only mild inflammation was seen in the gastric mucosa. Based on the abovementioned findings, the patient was diagnosed with PD, and intravenous DRPM (0.5 g twice a day) was started on day 5. After the initiation of this antibiotic therapy, the only effective antibiotic for these two strains (i.e., *S. parasanguinis* and *E. coli*) turned out to be carbapenems, including DRPM, according to antibiotic susceptibility tests. His high fever and abdominal symptoms gradually improved, and the C‐reactive protein level decreased to 0.52 mg/dl on day 18 (Figure 4). Although a repeat EGD on day 19 revealed that the reddish and edematous area extended to the gastric antrum and the second portion of the duodenum, the erosions and pus had disappeared. On day 20, the patient was discharged home in good condition. The thickened wall of the duodenum and the gastric antrum returned normal, which was confirmed by abdominal CT 2 months later.

## DISCUSSION

We herein report a rare case of PD, which was diagnosed based on the integrated medical findings, including a clinical course, biochemical and imaging examinations, a histopathological examination, and a culture of the duodenal mucosa. Despite the mucosal inflammation had subsequently extended to the adjacent stomach and the duodenal second portion, we speculate that the duodenal bulb was the epicenter of his infection on the basis of endoscopic and histopathological findings and that he was affected with PD under his immunocompromised state.

Among the types of phlegmonous infections in the digestive tract, the stomach is the most frequently affected organ, and this condition is well known as phlegmonous gastritis. It is an infectious disease of the gastric wall, which is caused by suppurative bacteria, such as *Streptococcus* spp., *Enterobacter* spp., *E. coli*, and *Proteus* spp.^1^ An immunocompromised state, including malignancy, chemotherapy, acquired immunodeficiency syndrome, alcohol addiction, diabetes mellitus, and immunosuppressive drugs, is considered the main predisposing factor.^2^, ^3^, ^4^ Abdominal pain, nausea, vomiting, and fever are the common symptoms of phlegmonous gastritis.^3^ Iqbal et al.^4^ reported that phlegmonous gastritis should be highly suspected if a CT scan reveals diffuse thickening of the stomach wall. In the absence of specific symptoms and signs of this disease, CT may provide an initial clue for the following diagnostic steps. Endoscopic findings of phlegmonous gastritis include edematous dark‐purple mucosa, friability, erosion, and purulent discharge.^5^, ^6^ Furthermore, cultures of the purulent discharge obtained using endoscopy not only provide a clue to identify the pathogens but can help guide the optimal antimicrobial selection. A typical pathologic finding of phlegmonous gastritis is neutrophil and plasma cell infiltration; however, it is sometimes nonspecific.^7^ The mortality rate of phlegmonous gastritis is reported to be 27%–40%,^4^ and this requires clinicians to manage it attentively using conservative antibiotic treatment or prompt surgical gastrectomy.

There is a paucity of reports on localized PD, and its clinical features and image findings have not been fully elucidated. Kneafsey et al.^8^ described a case of PD in a patient with multiple myeloma. Since this 58‐year‐old man developed acute abdominal pain and fever during chemotherapy, he underwent emergency laparotomy and was finally diagnosed with PD by *Streptococcus* spp. Garland et al.^9^ reported an autopsy case of PD in a 40‐year‐old man with alcoholic cirrhosis and diabetes mellitus. This patient died in the ambulance after he reported frequent vomiting for 1 day. Based on the autopsy findings, he was diagnosed with PD. Although these reports did not show the radiological and endoscopic findings, our case clearly presented the clinical features of CT and EGD. Moreover, the results of the microbiological examination obtained from the duodenal mucosa not only strengthened the diagnosis but identified the pathogens (*S. parasanguinis* and *E. coli*) and led to the treatment strategy. Our immunocompromised patient showed diffuse thickening of the duodenal wall on CT and the dark‐purple duodenal mucosa with purulent discharge on EGD. Our patient's background and clinical findings were similar to those of previously reported cases with phlegmonous gastritis. Therefore, this may suggest that clinicians can manage this uncommon disease based on accumulated knowledge about phlegmonous gastritis.

The etiology of PD in this case is worthy of discussion. Since the mucosal defect in the duodenal bulb was not a common duodenal ulcer, but rather appeared to be a fistula discharging pus from the deeper layer and there were no other infectious focuses in this patient, we consider this condition as idiopathic PD. Ishioka et al.^5^ indicated that most idiopathic cases with phlegmonous gastritis occur in immunocompromised hosts, as with our case. Taken together, we assume that circulating causative organisms from unknown entries settled in the duodenal submucosa under the vulnerable immunocompromised state.

In conclusion, although PD is an extremely rare condition, endoscopists should be aware of the specific endoscopic findings of this disease and obtain mucosal specimens for bacterial culture to establish the diagnosis and navigate further management. Only a high index of suspicion helps diagnose promptly and manage properly, particularly in immunocompromised patients.

## CONFLICT OF INTEREST

None.
